# The Role of *HOXB9* and *miR-196a* in Head and Neck Squamous Cell Carcinoma

**DOI:** 10.1371/journal.pone.0122285

**Published:** 2015-04-10

**Authors:** Lav Darda, Fahad Hakami, Richard Morgan, Craig Murdoch, Daniel W. Lambert, Keith D. Hunter

**Affiliations:** 1 Unit of Oral and Maxillofacial Pathology, School of Clinical Dentistry, University of Sheffield, Sheffield, United Kingdom; 2 Institute of Cancer Therapeutics, University of Bradford, Bradford, United Kingdom; 3 Unit of Oral and Maxillofacial Medicine & Surgery, School of Clinical Dentistry, University of Sheffield, Sheffield, United Kingdom; 4 Department of Oral Pathology and Biology, University of Pretoria, Pretoria, South Africa; Virginia Commonwealth University, UNITED STATES

## Abstract

**Background:**

Previous studies have demonstrated that a number of *HOX* genes, a family of transcription factors with key roles in early development, are up-regulated in head and neck squamous cell carcinoma (HNSCC) and other cancers. The loci of several Homeobox (*HOX*) genes also contain microRNAs (miRs), including miR-196a.

**Methods:**

Global miR expression and expression of all 39 *HOX* genes in normal oral keratinocytes (NOKs), oral pre-malignant (OPM) and HNSCC cells was assessed by expression microarray and qPCR and in tissues by immunohistochemistry (IHC) and qPCR of laser microdissected (LCM) tissues. Expression of miR196a and *HOXB9* was reduced using anti-miR-196a and siRNA, respectively. Expression microarray profiles of anti-miR196a and pre-miR196a transfected cells were compared to parental cells in order to identify novel targets of miR-196a. Putative miR196a targets were validated by qPCR and were confirmed as binding to the 3’UTR of miR196a by a dual luciferase reporter assay combined with mutational analysis of the miR-196a binding site.

**Results:**

miR-196a and *HOXB9* are highly expressed in HNSCC compared to NOKs, a pattern also seen in HNSCC tissues by HOXB9 IHC and qPCR of miR-196a in LCM tissue. Knock-down of miR-196a expression decreased HNSCC cell migration, invasion and adhesion to fibronectin, but had no effect on proliferation. Furthermore, knock-down of *HOXB9* expression decreased migration, invasion and proliferation but did not alter adhesion. We identified a novel primary mRNA transcript containing *HOXB9* and *miR196a-1* as predicted from *in-silico* analysis. Expression array analysis identified a number of miR196a targets, including *MAMDC2* and *HOXC8*. We confirmed that *MAMDC2* is a novel miR-196a target using a dual luciferase reporter assay with the effect abolished on mutation of the binding site.

**Conclusions:**

These results show that *miR-196a* and *HOXB9* are overexpressed, perhaps co-ordinately, as HNSCC develops and exert a pro-tumourigenic phenotype in HNSCC and OPM cells.

## Introduction

The identification of a number of key molecular alterations in cancer has resulted in major advances in diagnosis and targeted therapy with validated biomarkers, heralding the advent of personalised medicine. However, head and neck squamous cell carcinoma (HNSCC) lags behind, with no consistent oncogenic drivers identified and cetuximab being the only approved targeted therapeutic. This reflects both the molecular heterogeneity of this cancer and the paucity of understanding of its molecular landscape [1]. Worldwide, HNSCC presents a significant public health problem, being the 6^th^ most common cancer with survival rates which have not improved significantly for several decades [2]. Hence, there is a pressing need to find both novel targets for therapeutic intervention and new biomarkers in HNSCC.

Data mining of our published gene expression profile of normal, premalignant and HNSCC cells (http://bioinformatics.picr.man.ac.uk/vice/PublicProjects.vice?pager.offset=15 ) to identify deregulated pathways [3] has identified a number of consistently up-regulated transcription factors in HNSCC, including several Homeobox *(HOX)* genes (see Hunter et al, Supplementary data S3 and S4). *HOX* genes code for transcription factors with important roles in embryogenesis and organogenesis [4,5]. There are 39 *HOX* genes present on chromosomes 2, 7, 12 and 17, split into four clusters (A-D), and further sub-divided into 13 paralogous groups [4,6]. *HOX* proteins contain a 60 amino acid homeodomain that facilitates their binding to DNA [7]. *HOX* gene products interact with co-factors such as *PBX*, a member of the *TALE* family of homeodomain proteins, which alters their binding with DNA, regulates transcription and is needed for specific *HOX* functions [8].

*HOX* gene expression is dysregulated in many cancers, most significantly in leukaemia. In acute myeloid leukaemia (*AML)*, fusion proteins of *NUP98*:*HOXC11* and *NUP98*:*HOXD13* have been identified which result in aberrant HOX trans-regulatory activity [9,10]. In breast cancer, *HOXA5* and *HOXB13* expression is down-regulated [11,12] whereas *HOXB9* is highly up-regulated [13] and changes in *HOX* gene expression have been reported in lung [14,15] and gastric cancer [16]. In HNSCC, several *HOX* genes show higher levels of expression in pre-malignant and cancer tissues compared to normal tissues [17]. Overall, 18 *HOX* genes were more highly expressed in HNSCC cells than in normal cells, among them *HOXB9*. However there is a lack of clarity in the literature as to the extent and relative importance of *HOX* genes in HNSCC carcinogenesis.

*HOX* clusters also contain microRNAs; non-coding RNA transcripts which bind predominantly to the 3’UTR of target transcripts [18–21], resulting in translational repression or degradation of the mRNA transcript [19]. MicroRNA (miR)-196 is present in three *HOX* clusters: miR-196b on 7p15 (*HOXA*), miR-196a-1 on 17q21 (*HOXB*) and miR-196a-2 on 12q13 (*HOXC*). miR-196a-1 and miR-196a-2 have an identical mature sequence, whereas miR-196b differs by a single nucleotide [22]. These miRNAs target *HOX* genes located 5’ of their locus, supporting the theory of posterior prevalence [21,23]. miR-196a targets several *HOX* genes, including *HOXA5*, *HOXB7*, *HOXB8* and *HOXC8* [18,19,24,25] and has also been shown to directly target several other genes such as *ANXA1*, *p27*, *KRT5*, *S100A9* and *SPRR2C* [20,26,27]. Expression of miR-196a is up-regulated in breast, gastric, lung and oesophageal cancers [16,20,25,28], whereas it is down-regulated in melanoma [29]. miR-196a has been shown to be up-regulated in HNSCC and may also be detected in the serum of these patients pre-operatively [22,30]. In a recent meta-analysis of miR profiling in HNSCC tissues, miR196a was identified, but only in a minority of the studies assessed. Severino *et al* have shown that transfection of miR196a into normal oral keratinocytes decreases proliferation, but with no effect on the expression of previously described miR196a targets [30]. Thus, the functional consequences of miR-196a alteration in HNSCC and the protein-coding targets mediating any phenotypic changes remain to be fully determined.

In this study, we show that miR-196a and *HOXB9* are the most markedly differentially expressed miR and *HOX* gene respectively when comparing HNSCC and NOKs. This up-regulation was also seen in head and neck cancer tissue compared to normal tissue and bioinformatics analysis suggests that these may be co-expressed (Ensembl Transcript: RP11-357H14.19–001). Thus, we aimed to evaluate the functional effects of high miR196a and HOXB9 expression in HNSCC and to identify novel and direct targets of miR-196a in this malignancy.

## Materials and Methods

### Cell culture and tissue samples

B16, B22, B56 (BICR56), T4 (HNSCC cell lines, Beatson Institute for Cancer Research [31,32]), H357 (HNSCC cell line from Prof S Prime [33]), D19, D20, D4, D35 (oral pre-malignant (OPM) cell lines, Beatson Institute for Cancer Research[34,35]), primary normal oral keratinocytes (NOKs; isolated as previously described [36], grown with irradiated 3T3 cells), and OKF4 (immortalized normal oral keratinocytes; iNOK; from J Rheinwald, Boston, USA[37]) were maintained in keratinocyte growth medium (KGM) consisting of DMEM supplemented with 23% Ham’s F-12, 10% FCS, L-glutamine (2mM), adenine (0.18mM), hydrocortisone (0.5μg/ml) and insulin (5μg/ml) (all Sigma Aldrich, Poole, UK). CAL27 (HNSCC cell line, ATCC) was maintained in High glucose containing DMEM supplemented with 10% FCS and L-glutamine (2mM). All cell lines were incubated at 37°C and 5% CO_2_. None of the cell lines used had a published STR profile, thus we conducted baseline STR profiling to assure no similarity with any other cell line with a known profile. This was conducted immediately prior to the experiments described below. Further information of the cells lines used in this study can be found in S1 Table.

A tissue microarray consisting of 25 oral cavity HNSCCs was available for HOXB9 immunohistochemistry. The clinicopathological details of this cohort are presented in S2 Table. Three cores from the body of the tumour and 3 cores from the advancing edge were available for each sample. A cohort of normal oral mucosal biopsies was used for comparison, with a site distribution matching the HNSCC cohort. A further cohort of 16 HNSCC (S3 Table) with site matched normal oral mucosa (from different patients) was used for the assessment of miR196a expression in tissues.

### RNA isolation and quantitative RT-PCR

Total RNA was extracted using the RNeasy mini kit (Qiagen, Manchester, UK) and quantified using a NanoDrop Spectrophotometer (Thermo Scientific, Hemel Hempstead, UK). High capacity RNA-to-cDNA kit (Life Technologies, Paisley, UK) was used for cDNA synthesis from total RNA. For miRNA cDNA synthesis, 10ng RNA was reverse transcribed using miRNA-specific primers. For total cDNA synthesis, 200ng RNA was reverse transcribed using random primers. Quantitative gene expression analysis was performed using SYBR® green or Taqman with appropriate controls (U6 for SYBR and RNU48 (Taqman ID: 001006) for Taqman analysis; hsa-miR-196a (Taqman ID: 241070)). Initial screening for all HOX genes was by SYBR green qPCR on a Stratagene platform MX3005p (primers sequences in S4 Table) [14]. Additional qPCR was performed using an ABI 7900HT (Life Technologies, Paisley, UK) and the relative expression of genes, normalised to the abundance of the relevant control transcript, was calculated using RQ Manager 1.2.1 (Life Technologies, Paisley, UK).

### Affymetrix miRNA array

RNA from duplicate cultures of NOK (normal OKs), Cal27 and BICR56 (both HNSCC) extracted using the mirVana miRNA isolation kit (Life Technologies, Paisley, UK) and was then prepared as per the manufacturer’s protocols (http://media.affymetrix.com/support/downloads/manuals/flashtag_user_guide.pdf) prior to hybridisation onto the Affymetrix GeneChip miRNA 1.0 Array. The array data was normalised using RMA (RMAEXpress) and then loaded into tMeV (Craig Venter Institute, Rockville, CA) and analysed using the statistical analysis of microarray tool (SAM) with a false discovery rate <1% and fold change of >5, comparing NOK with HNSCC. The primary data is available in the NCBI GEO database (accession number GSE52811).

### *HOXB9*-miR-196a-1 primary transcript

To investigate the presence of a common *HOXB9*-miR-196a-1 primary transcript (PT), primers were designed that spanned the 6.3Kb between *HOXB9* exon1 and miR-196a-1 precursor transcript (forward: 5’ AATTAGGTAGTTTCATGTTGTTGGGCC 3’; reverse: 5’ ATAATAGCTGCTAAGCGTCCC AGAAAT 3’). For the reverse transcription step, primary transcript primers were used with M-MLV reverse transcriptase (Promega, Southampton, UK), followed by PCR using the same primers (product 6.3kB). Nested primers were designed to give a 295bp product within this transcript (forward: 5’ AAAGTCAGGGCAGGAGAGGGAAGGGGAA 3’, reverse: 5’ CAATTTGCCAGCCCTATGAAGTCTGCT 3’), with RNaseA treated (RNA was incubated for 1 hour at 37°C with 100μg/ml RNaseA (Promega, Southampton, UK) and no-reverse transcriptase (RT) controls. The PCR products were separated on a 2% (w/v) agarose gel, visualised under UV transillumination and purified using gel extraction kit (Bioline, London, UK). This product was then cloned into TOPO TA Cloning vector (Life Technologies, Paisley, UK) and positive colonies were selected using blue/white screening, purified using Isolate plasmid mini kit (Bioline, London, UK) and sequenced.

### Laser Capture Microdissection (LCM)

Fresh 4μM sections of formalin-fixed, paraffin-embedded (FFPE) tumour and normal tissue (as described above) were de-waxed, stained with haematoxylin and dehydrated by xylene. LCM was carried out using a Pixcell II LCM system (Arcturus, Life Technologies, Paisley, UK). RNAqueous® Micro kit (Life Technologies, Paisley, UK) was used to extract RNA from the cells, according to the manufacturer’s instructions.

### Protein extraction and western blotting

RIPA buffer (Sigma Aldrich, Poole, UK) containing protease and phosphatase inhibitors (Roche, West Sussex, UK) was added to the cell pellet on ice and then centrifuged at 13,000rpm for 5 min at 4°C. The protein was quantified using BCA method [38] as per manufacturer’s protocol (Thermo Scientific, Hemel Hempstead, UK). 40 μg of total protein was loaded onto a 12% (v/v) SDS-PAGE gel. Wet transfer for 1h at 30V transferred protein to a nitrocellulose membrane, before incubation for 1h in blocking buffer (5% (w/v) dried milk with 3% (w/v) BSA in tris buffered saline containing 0.05% (v/v) tween-20) and incubation overnight at 4°C with the primary monoclonal antibody anti-HOXB9 (1:500 in blocking buffer, Abcam, Cambridge, UK) or anti-β-actin (1:3,000 in blocking buffer, Sigma Aldrich, Poole, UK). The membrane was then incubated in horseradish peroxidase-conjugated anti-rabbit IgG (1:3000 in blocking buffer) for 1h and developed with SuperSignal West Pico chemiluminescent substrate (Thermo Scientific, Hemel Hempstead, UK).

### Immunohistochemistry (IHC)

Antigen retrieval was performed using a pressure cooker [39] on sections followed by incubation with anti-*HOXB9* (1: 400, Sigma Aldrich, Poole, UK) overnight at 4°C. Vectastain Elite ABC rabbit IgG kit (Vector Laboratories Inc. Burlingame, CA, USA) was used for secondary antibody (30min at room temperature), followed by colour development by DAB reagent (Vector Laboratories Inc. Burlingame, CA, USA). Submandibular salivary gland and sections with no primary antibody were used as positive and negative controls respectively. IHC was analysed by the semi-quantitative modified Quickscore method on 6 cores from each tumour [40].

### Anti-miR and siRNA transfection

B16, D19 or OKF4 cells were seeded in a 6-well plate and incubated overnight. Keratinocytes were transfected when 50–70% confluent with anti-miR-196a or pre-miR-196a (50nM, Life Technologies, Paisley, UK) or siRNA targeting human *HOXB9* (50nM, Sigma Aldrich, Poole, UK) using Oligofectamine (Life Technologies, Paisley, UK) according to the manufacturer’s protocol. A random, non-targeting negative control sequence (Life Technologies, Paisley, UK) was used in all experiments. Cells were transfected and incubated for 48h in DMEM supplemented with 20% FCS before being used for the functional assays.

### Proliferation Assay

Cells were seeded in a 96-well plate (Corning Inc, Corning, NY, USA) in triplicate for each time point (0, 24, 48, 72 and 96h) at a density of 5x10^3^ cells in DMEM media supplemented with 10% (v/v) FCS. MTS reagent (Promega, Southampton, UK) was added to the wells, incubated for 1h and then read at 490nm on a Tecan Infinite M200 spectrophotometer (Tecan, Männedorf, Switzerland) using Magellan software.

### Adhesion Assay

A 96-well plate was coated with 0.1% (w/v) fibronectin (Sigma Aldrich, Poole, UK) in PBS (1:100) and incubated overnight at 4°C. The following day wells were washed with PBS and incubated with DMEM containing 1% (w/v) BSA for an hour. The transfected cells were plated in triplicate at a density of 3x10^4^ cells/well in DMEM and incubated for 1h. Non-adherent cells were removed by washing with PBS. MTS reagent (Promega, Southampton, UK) was added to the wells, incubated for 1h and then read at 490nm on a Tecan Infinite M200 spectrophotometer.

### Transwell Migration and Invasion Assay

To assess migration, transfected cells (8x10^4^) in DMEM with 0.1% (w/v) BSA were placed, in duplicate, in the top chamber of a 24-well transwell insert (8μM pore size; BD Biosciences, Oxford, UK). The bottom of the 24-well plate was filled with DMEM supplemented with 2% (v/v) FCS and the cells incubated overnight at 37°C. For invasion assays the top chamber of a transwell insert was covered with 100μL of growth factor reduced matrigel (BD Biosciences, Oxford, UK) and incubated overnight at 37°C. 8x10^4^ transfected cells in 0.1% (w/v) BSA in DMEM were placed in the top chamber in duplicate. DMEM containing 2% (v/v) FCS was placed in the bottom of the 24-well plate followed by 48h incubation at 37°C. Mitomycin C (1μg/mL) was added to the medium in both chambers. For both assays the migrating or invading cells present on the underside of the transwell insert were stained with crystal violet and then counted at four random fields by light microscopy.

### Agilent Gene expression microarray

The quality of total RNA was assessed using an Agilent Bioanalyzer, (Agilent, Wokingham, UK) with QC thresholds 28s:18s ≥2:1 and RIN = 10. Biological triplicates of B16 and D19 cells were transfected with anti-miR-196a and negative control, whereas OKF4 cells were transfected with pre-miR-196a and negative control in duplicate (total 16 samples). Samples were prepared and hybridised to Agilent oligonucleotide microarray chips (Sureprint G3; Agilent, Wokingham, UK) according to the manufacturer’s protocol (http://www.chem.agilent.com/library/usermanuals/public/g4140-90041_one-color_tecan.pdf). Data was loaded into Qlucore Omics Explorer (Qlucore AB, Lund, Sweden) and, following normalisation and principle component analysis, T-test (p<0.01, with multiple test correction) was used to compare the transfected with the parental cells for each cell line. The top 50 up- and down-regulated genes were selected for further validation by qPCR. The primary data is available in the NCBI GEO database (accession number GSE52810).

### Luciferase reporter assay and site directed mutagenesis

The *MAMDC2* 3’UTR was amplified using cDNA from B16 cells (primers: wt forward 5’ AAAAAAAAA CGCGTAAATGATCTGCATTGGATTTACT 3’ and wt reverse 5’ AAAAAAAAGTTTAAACAAGATTTT CAAATTATTTTTATTAGGTAATTTTATAATTTC 3’ containing MluI and PmeI restriction sites, respectively). The amplified PCR product was ligated into pMIR-REPORT (Ambion). The miR-196a binding site within the *MAMDC2* 3’UTR was mutated using PCR-based site-directed mutagenesis. pMIR REPORT cloned with wt 3’UTR was used as the template in the PCR. The primers used for mutation of the miR-196a binding site had T_m_ of > = 78°C (*MAMDC2* mutant forward: 5’ CCTTCTTTA TTCCCCCTTTGA**GACG**C**T**TTTGAAGTCACTATAGC 3’ and MAMDC2 mutant reverse: 5’ GCTCAT AGTGATTCAAA**A**G**CGTC**TCAAAGGGGGAATAAAGAAGG 3’, mutated bases in bold). The PCR product was incubated for 1h with DpnI (New England Biolabs, Herts, UK) to degrade methylated template plasmid. 500 ng of pMIR vector (wt or mutant) was transfected into B16 HNSCC cells with 50 ng of pRL-TK Renilla luciferase vector (Promega, Southampton, UK) and pre-miR-196a or scrambled negative control (50nM) (Life Technologies, Paisley, UK) using Fugene HD transfection reagent (Promega, Southampton, UK). The cells were incubated for 48h and then the luciferase activity for firefly and renilla was measured using dual luciferase reporter assay (DLRA) (Promega, Southampton, UK) according to the manufacturer’s protocol. The assay was repeated thrice in triplicate.

### Statistical Analysis

Non-parametric Mann-Whitney U test was performed on the IHC tissue sample scoring for HOXB9 and the absolute value of the qPCR performed on LCM tissue sample for miR-196a. Parametric student’s t-test was used to calculate all other significance values. Results were only considered significant if p<0.05.

### Ethics Approval

Ethics approval for the use of biopsy tissues in this study was obtained from The West Glasgow LREC (ref: 08/S0709/70). Ethical approval for the normal oral keratinocyte primary cultures used was obtained from the National Research Ethics Service, UK: 09/H1308/66 and human tissue was used with written, informed consent. The Ethics Approval waived the need for specific consent in both cases as the material to be used was anonymised and surplus to diagnostic requirements.

## Results

### miRNA microarray analysis reveals high miR-196a expression in HNSCC-derived cell line

**Fig 1:**
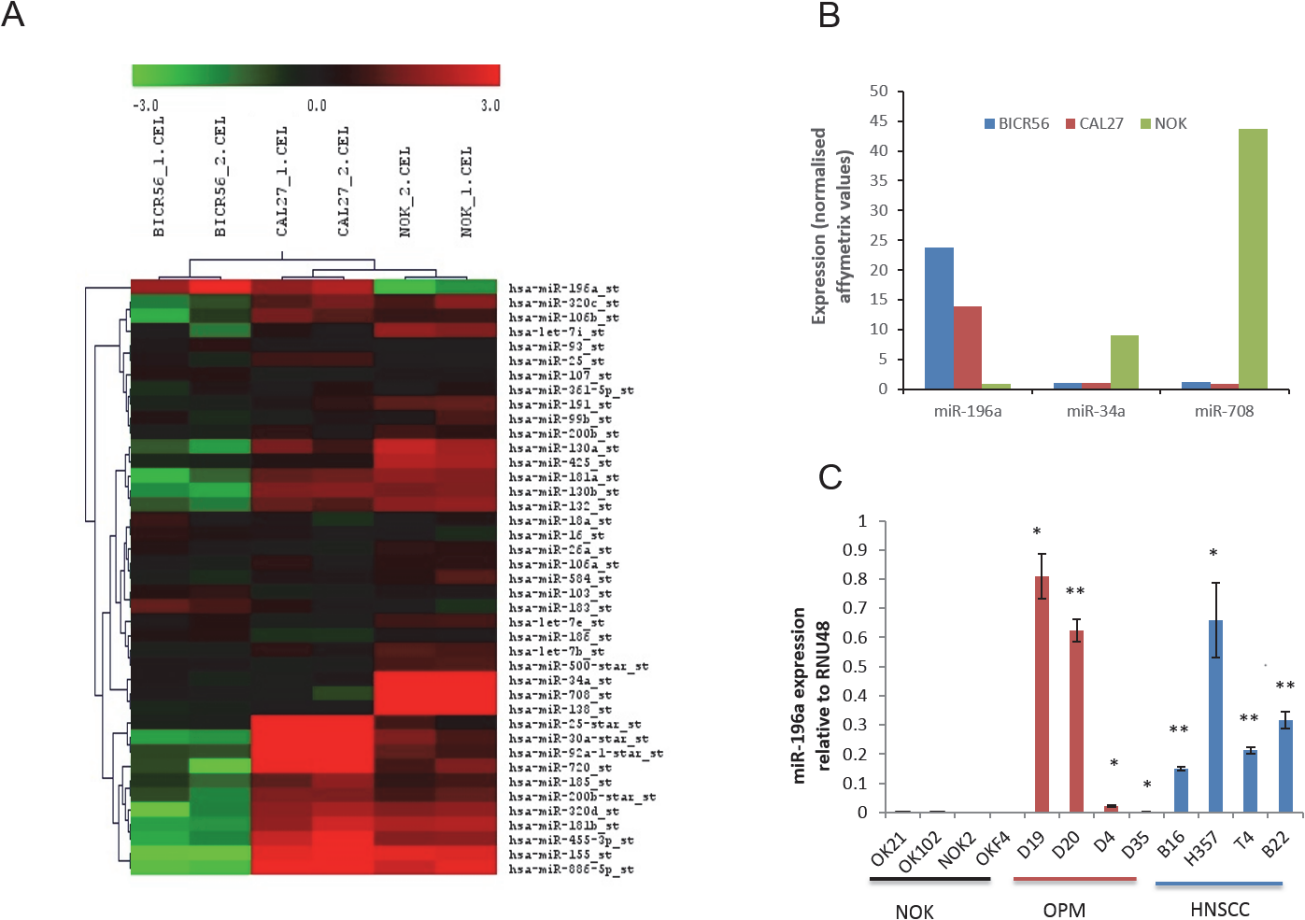
miR-196a is up-regulated in OPM and HNSCC-derived cell lines. **1A:** Heat map showing differentially expressed microRNAs in microarray data from Affymetrix miRNA array on comparison of HNSCC and NOK using SAM with a FDR of 1%. BICR56 and CAL27 are HNSCC cells and NOKs are normal oral keratinocytes. The values presented are the mean of 2 technical replicates. **1B**. Candidate miRs were selected as differentially expressed with a FDR of 1% and fold change ≥5. **1C**. Expression of miR-196a in a panel of NOK, OPM and HNSCC cells. * p<0.05, ** p<0.01.

MicroRNA profiling using the Affymetrix miRNA array identified a number of significantly differentially expressed miRs between the NOKs and HNSCC cells (Fig 1A). As the number of samples was small the stringency applied in analysis was high (FDR<1%), in order to robustly identify differentially expressed miRs for further analysis. The number of hits was further limited to those that changed ≥5 fold, finally identifying miR-196a, miR-34a and miR-708, as being differentially expressed (Fig 1B). Of these, only miR-196a was highly expressed in HNSCC. Validation of miR-196a expression by qPCR in a full panel of NOK, OPM and HNSCC cells showed high expression in all HNSCC cells tested with variable expression in OPM cells, two of which had similar expression to that seen in the HNSCCs (Fig 1C). Expression in NOKs was consistently low.

### *HOXB9* is the most highly expressed of a number of HOX genes elevated in HNSCC-derived cell lines

**Fig 2:**
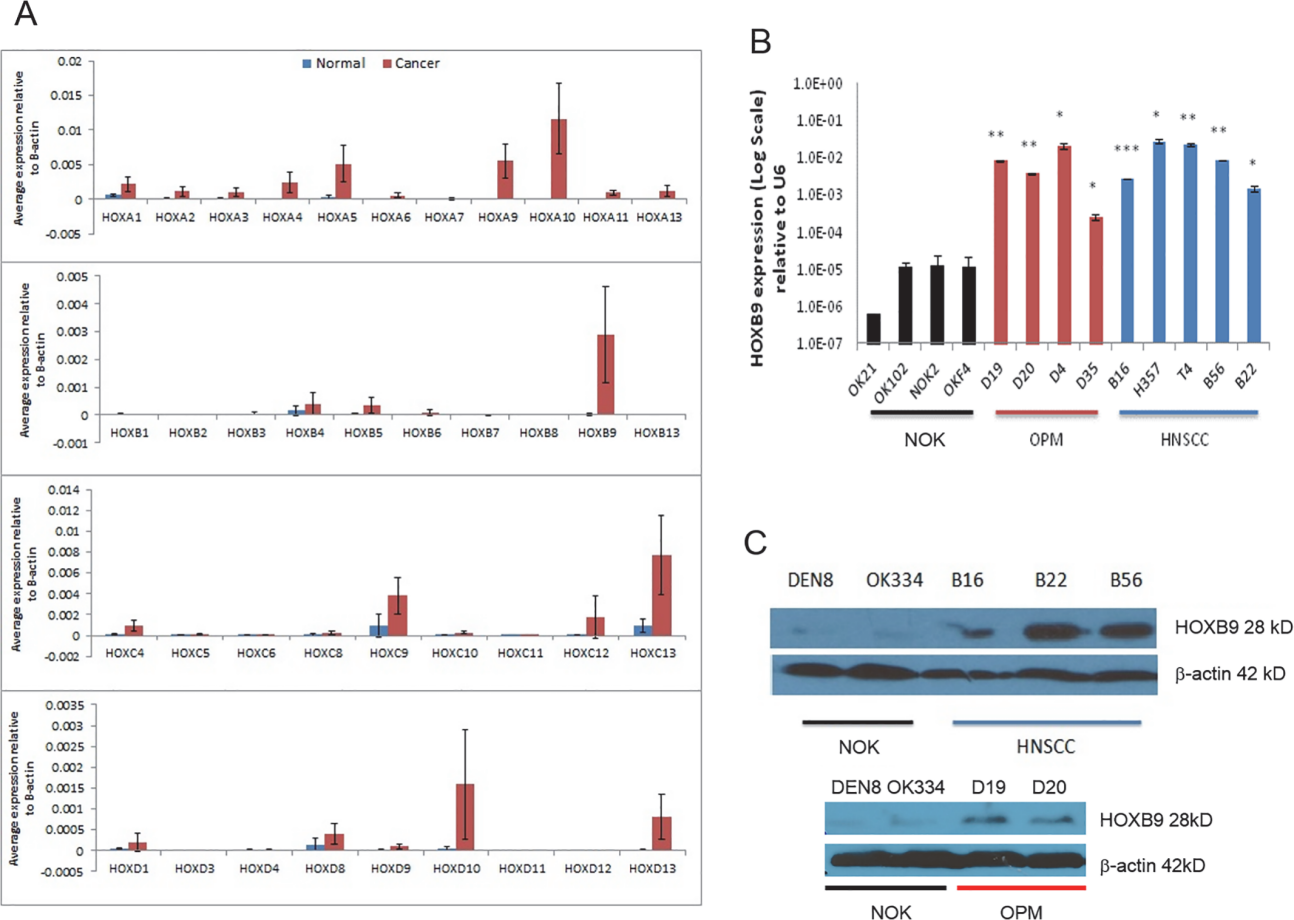
*HOXB9* is over-expressed in OPM and HNSCC-derived cell lines. **2A.** Overall mean expression of all 39 *HOX* genes as measured by qPCR in a panel of normal oral keratinocytes and oral cancer cells, relative to the internal endogenous control *β-actin*. In general, there is increased expression of most *HOX* genes, but particularly *A4*, *A10*, *B9* and *D10*. **2B.** Expression of *HOXB9* in a panel of NOK, OPM and HNSCC cells. **2C.** Expression of *HOXB9* protein assayed by Western blotting for NOK cells compared to HNSCC (upper panel) and OPL (lower panel). * p<0.05, ** p<0.01, *** p<0.001.

Expression analysis of all 39 *HOX* genes revealed that all were more highly expressed in HNSCC compared to NOK (Fig 2A and S2 Fig). The highest differential expression seen was of *HOXA4* (291 fold, p<0.01), *HOXA5* (105 fold, p<0.01), *HOXA9* (155 fold, p<0.001), *HOXB9* (1293 fold, p<0.001), *HOXC9* (41 fold, p<0.01) *and HOXD10* (23 fold, p<0.01), all of which are well recognised in the literature [7,17,41,42], but there was marked variability in the extent of the fold change. *HOXB9* was the most markedly differentially expressed, on average >1000-fold higher than in NOK. This was validated by qPCR, which demonstrated consistently high gene expression of *HOXB9* in OPM and HNSCC cells (Fig 2B). Despite a lack of direct correspondence in HOXB9 protein levels (particularly for B16), the difference in expression was also seen in Western blot, with the HNSCCs and OPMs expressing more HOXB9 protein than NOK, (Fig 2C).

### miR-196a and HOXB9 are more highly expressed in HNSCC tissues than in normal oral mucosa

**Fig 3:**
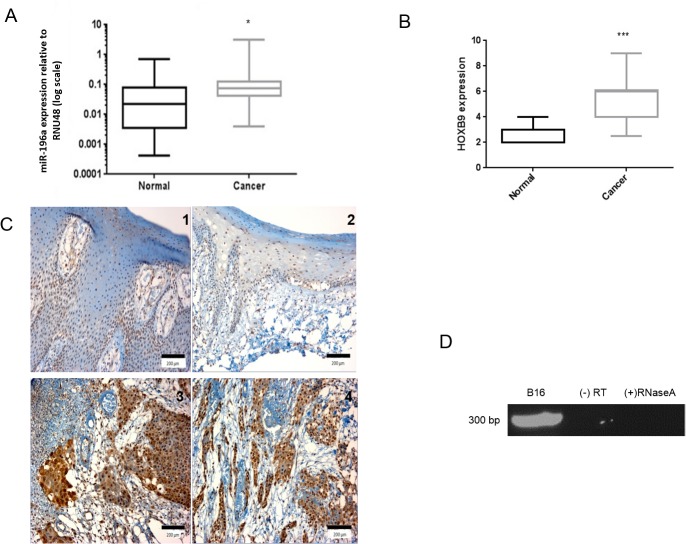
miR-196a and *HOXB9* are up-regulated in HNSCC tissue samples. **3A-C**. Expression of miR-196a (3A) and *HOXB9* in tissues (3B, 3C) measured by qPCR of LCM tissues and immunohistochemistry, respectively. Representative photomicrographs show HOXB9 expression in normal tissue (C panels 1 and 2) and HNSCC (C panels 3 and 4). The *HOXB9* expression in tissue was semi-quantitatively assessed using the Quickscore method. The pattern of expression correlates with *in-vitro* data and both are up-regulated in cancer tissue, albeit the fold change is less (3.4 fold for miR-196a). * p<0.05, ***p<0.001. Photomicrographs overall magnification x200, scale bar = 200μm. **3D.** miR-196a-1 and *HOXB9* are co-expressed on same novel primary transcript DNA agarose gel electrophoresis showing presence of 295 bp transcript after nested PCR. The transcript is present in B16 cell line and at the expected size. (-) RT: without reverse transcriptase control, (+) RNaseA: RNA treated with RNaseA at 37°C for 1 hour before cDNA production.

The expression of miR-196a in tissues was assessed by qPCR of RNA extracted from laser-captured FFPE tissue. Whilst RNA from FFPE tissues is often degraded, the small size of microRNAs allows efficient recovery from fixed tissue [43]. Significantly higher expression of miR-196a was observed in 16 HNSCC tissues when compared to 16 unmatched normal mucosa (Fig 3A, p<0.05), with some variation in expression seen in the normal samples. Analysis of HOXB9 expression by immunohistochemistry in a TMA containing 25 HNSCC samples compared with 10 normal oral mucosa samples revealed that this protein was more highly expressed in HNSCC compared to normal samples, as assessed by the Quickscore method (Fig 3B and 3C, p<0.001). In normal tissues, expression of *HOXB9* was expressed at low levels, confined to the nuclei of the basal and spinous layers (Fig 3C Panel 1 and 2), whilst in HNSCC the nuclear intensity increased, with a greater proportion of cells in tumour nests expressing *HOXB9* (Fig 3C Panel 3 and 4). Furthermore, in the HNSCC tissues, there was cytoplasmic expression of *HOXB9* in many cells.

### *HOXB9* and miR-196a-1 are co-transcribed on the same novel primary transcript

*HOXB9* and miR-196a-1 are spatially closely related on Chr17. To show that *HOXB9* and miR-196a-1 are co-transcribed on same primary transcript, primers were designed which spanned between *HOXB9* exon 1 to miR-196a-1 precursor transcript (6.3 Kb). Nested primers were designed which amplified a 295bp region within this 6.3 Kb transcript (S1 Fig). A product of the expected molecular size was obtained (Fig 3D); no products were observed in the absence of reverse transcriptase (-RT) or following prolonged incubation with RNase A, suggesting this product is derived from a *HOXB9*-miR-196a-1 polycistronic primary transcript, rather than from genomic DNA contamination.

### *HOXB9* and miR-196a increase HNSCC cell migration and invasion

**Fig 4:**
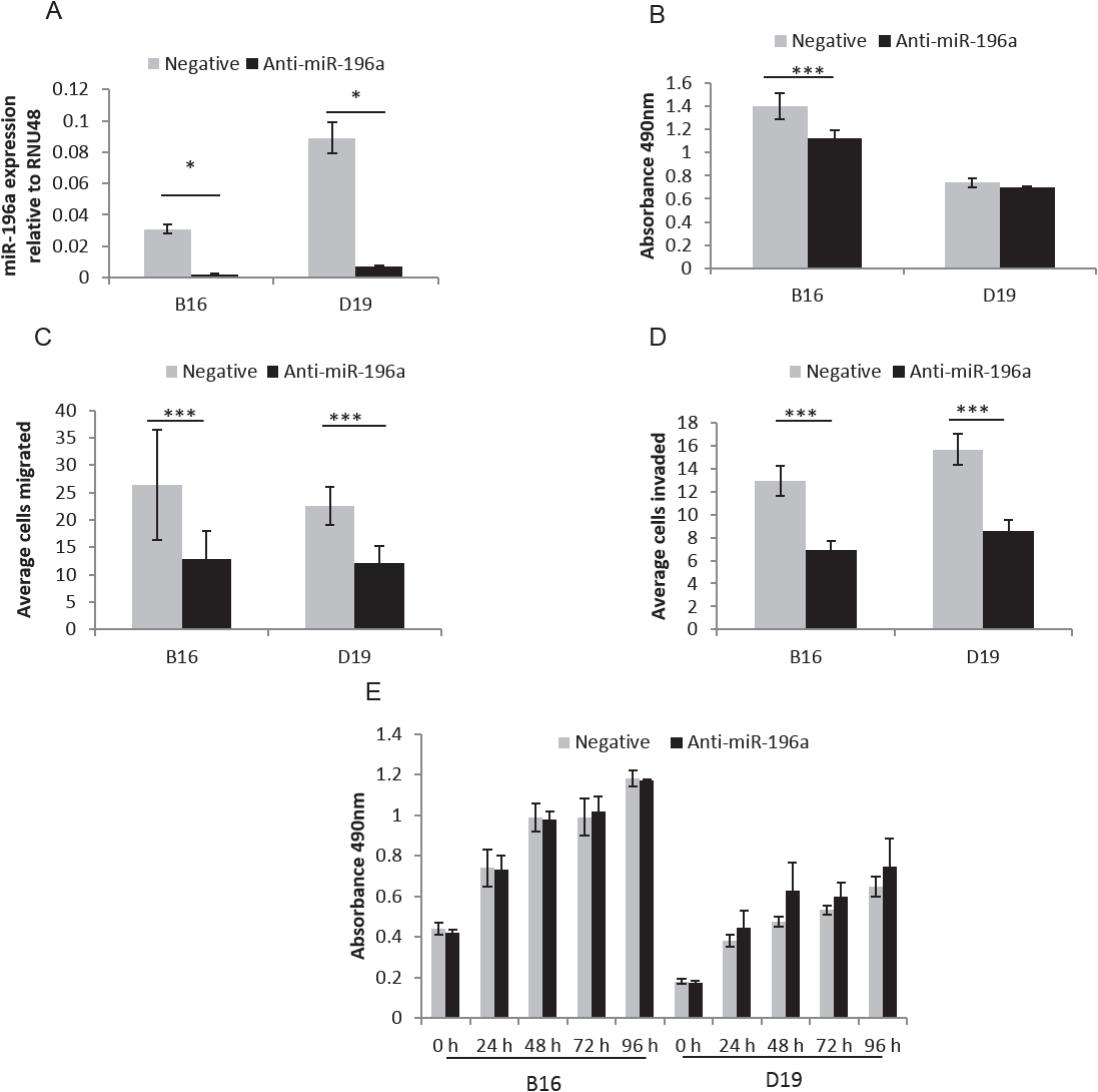
Functional effects of anti-miR-196a in HNSCC-derived cell lines. **4A.** B16 and D19 cells were transfected with anti-miR-196a and negative control resulting in 95% and 92% reduction in miR-196a expression respectively compared to negative control. **4B-E.** Anti-miR-196a decreases adhesion to fibronectin (B; in B16 only), migration (C), invasion (D) but has no effect on proliferation (E) in HNSCC cells. * p<0.05, *** p<0.001. All experiments were conducted in duplicate and completed three times.

**Fig 5:**
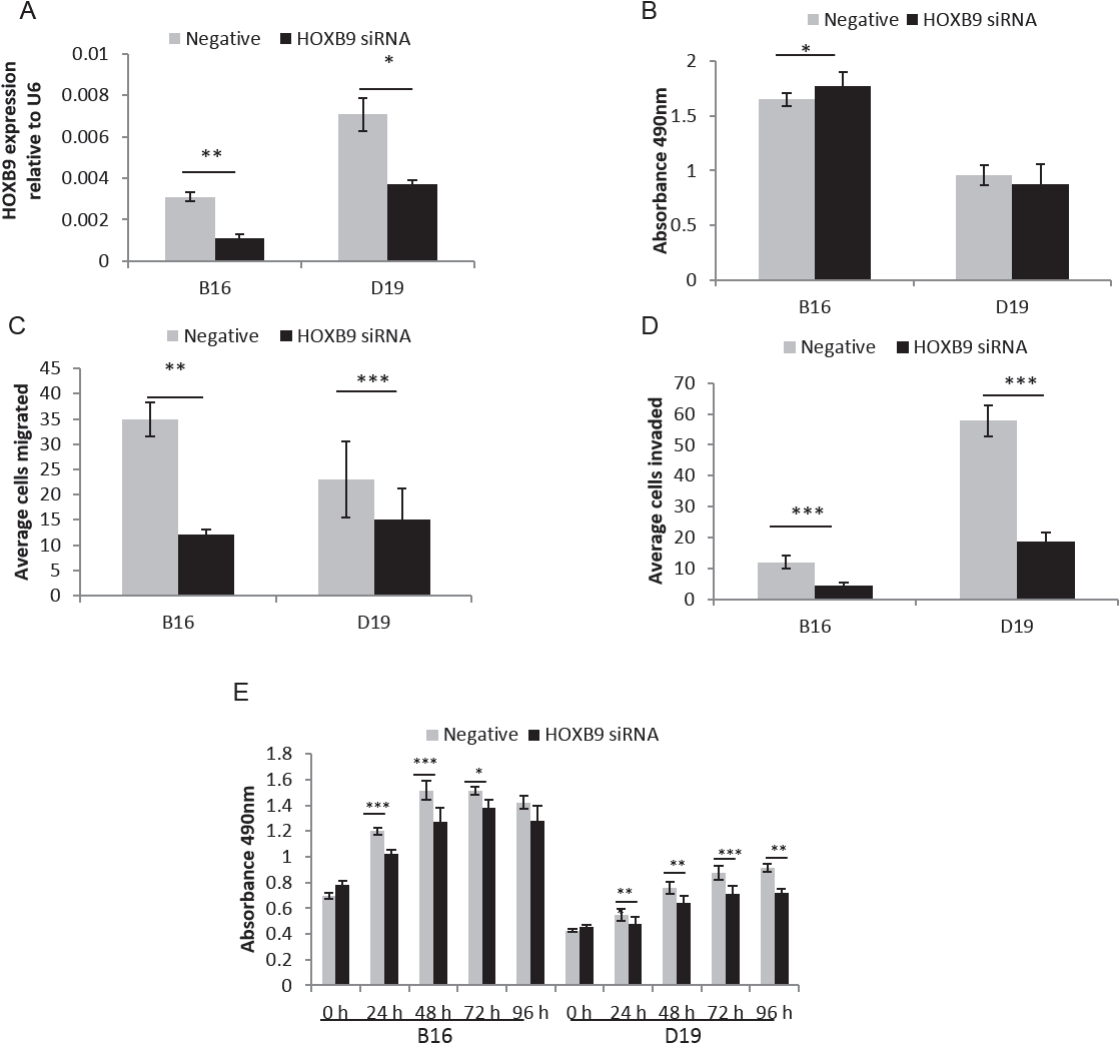
Functional effects of *HOXB9* siRNA in HNSCC-derived cell lines. **5A**. B16 and D19 cells were transfected with *HOXB9* siRNA and negative control. There was 62% and 48% down-regulation seen in *HOXB9* expression compared to negative control in B16 and D19 respectively. **5B-E.** *HOXB9* siRNA decreases migration (C), invasion (D) and proliferation (E) with no consistent change in adhesion to fibronectin (B) in HNSCC cells. *p<0.05, **p<0.01, ***p<0.001. All experiments were conducted in duplicate and completed three times.

Given that both *HOXB9* and miR-196a are highly expressed in HNSCC we assessed the phenotypic consequences of this in OPM and HNSCC cells, particularly assessing features that promote the ability of the cells to proliferate and to spread, enhancing the development of the tumour. Reducing expression of miR-196a or *HOXB9* by anti-miR-196a (95% (B16) and 92% (D19)) and HOXB9 siRNA (62% (B16) and 48% (D19)), respectively (Figs 4A and 5A), reduced the ability of the OPM and HNSCC cells to migrate and invade into Matrigel (Figs 4C, 4D, 5C and 5D). The effects on proliferation and adhesion to fibronectin, a major component of the extracellular matrix, were more variable as reducing miR-196a expression reduced adhesion in HNSCC cells but not OPM cells, with no effect on proliferation in either cell type (Fig 4B and 4E). Supporting these observations, increased migration was seen after transfection of OKF4 (immortalised NOK) with pre-miR-196a, whilst there was no effect on proliferation or adhesion (data not shown). Reducing *HOXB9* expression by siRNA additionally reduced proliferation of OPM and HNSCC cells (Fig 5E). Adhesion to fibronectin was significantly increased in B16 only, but only to a small degree (Fig 5B).

### Expression microarray analysis identifies known and putative miR-196a targets

**Fig 6:**
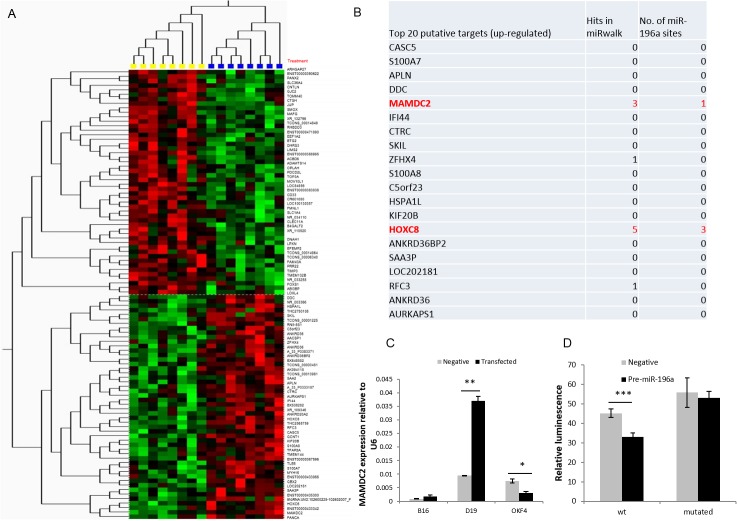
Prediction of putative targets of miR-196a in HNSCC-derived cell lines. **6A.** Microarray Heat Map depicting top 50 miR-196a up-regulated and down-regulated genes. Yellow samples were compared against blue samples (high and low miR-196a expression respectively) using Qlucore Omics Explorer with T-test, p<0.01. Yellow box consists of B16 negative control, D19 negative control and OKF4 pre-miR-196a transfected cells whereas blue box consists of B16 anti-miR-196a, D19 anti-miR-196a and OKF4 negative control transfected cells. **6B**. Table depicting top 20 up-regulated putative targets based on expression analysis and miRwalk analysis of potential miRNA targets. It also shows number of miR-196a binding sites present in 3’UTR of the gene (www.microRNA.org) **6C:** The changes in *MAMDC2* expression on transfection of anti-miR-196a were validated by qPCR in samples used in microarray. **6D**: pMIR-REPORT luciferase vector was cloned with wild-type *MAMDC2* 3’UTR (wt) and the predicted miR-196a site in the 3’UTR was mutated by site-directed mutagenesis (mutated). These were co-transfected with negative control or pre-miR-196a, pRL-TK renilla luciferase control vector into B16 (HNSCC cells). The relative luminescence value (firefly/renilla value) of wt/pre-miR-196a was significantly lower than wt/negative control, with no significant difference in the mutated 3’UTR. *p<0.05, **p<0.01, ***p<0.001.

Expression array data from both anti-miR (B16 and D19) and pre-miR (OKF4) transfected cells were used to identify consistent changes in gene expression related to alterations in miR-196a expression. This approach identified 353 altered genes (p<0.01 by t-test) with the top 50 up- and down-regulated shown in Fig 6A. Gene Ontology (GO) enrichment analysis using DAVID (http://david.abcc.ncifcrf.gov) demonstrated a number of over-represented GO biological processes, including amine/amino acid transport, DNA repair and regulation of transcription (S5 Table). From this list, the top 20 up-regulated genes underwent further *in-silico* analysis for predicted miR-196a binding to the 3’UTR of each gene, to further focus the list to potential direct targets of miR-196a (Fig 6B). Other than HOXC8 (S3D Fig), which has already been demonstrated to be a target of miR196a [18], the gene with the highest predicted interaction with miR-196a was MAM Domain Containing 2 (*MAMDC2*), which showed a good match based on sequence complementarity, energy of binding and evolutionary conservation of the site of 3’UTR to miR-196a (www.microrna.org). qPCR analysis of a number of reported miR196a targets in other cancers, *KRT5*, *ANXA1*, *S100A9* and *HOXC8*, was conducted (S2 Fig) [20,27]. These showed no consistent change on knockdown of miR196a in HNSCC and OPM cells, although *HOXC8* was differentially expressed in anti-miR196a transfected D19 cells.

### *MAMDC2* is a novel direct target of miR-196a

The change in *MAMDC2* expression seen on manipulation of miR-196a expression was validated by qPCR in the cells used in the microarray (Fig 6C). This confirmed the microarray data, showing *MAMDC2* expression increased after anti-miR-196a transfection in OPM (p<0.01) and HNSCC (p = 0.08) cells and was reduced after pre-miR-196a transfection into immortalised NOK (p<0.05). miR-196a over-expression significantly suppressed luciferase activity from a wild-type *MAMDC2* 3’UTR reporter construct in B16 cells (Fig 6D). This suppression was not observed following mutation of the predicted miR-196a binding site (Fig 6D), indicating a direct effect of miR-196a on *MAMDC2* expression at a transcript level.

## Discussion

There is now considerable evidence that miR-196a and HOXB9 exert a pro-tumorigenic influence in many cancers [13,15,22,27]. Our data convincingly demonstrate high expression of both miR-196a and *HOXB9* in HNSCC and in at least a subset of OPMs. This is in keeping with recent studies in HNSCC and also a number of other cancers, including breast and non-small cell lung carcinoma (NSCLC) [5,7,22,28,30,44]. Furthermore, a key role for miR-196a polymorphisms has emerged in relation to cancer risk, conferring increased susceptibility to a number of cancers, particularly in Asian populations [45]. Despite a well-recognised role in invasive disease, the function of miR-196a overexpression in the pre-invasive stage of HNSCC development has not been investigated before. We found high miR-196a expression in some OPM samples, whilst in others the level of expression is similar to NOK. Further investigations will be required to define the role of this overexpression and whether OPMs with high miR-196a expression progress to HNSCC.

Recent investigations in oral carcinomas have also demonstrated a pro-tumorigenic phenotype in cells expressing high levels of miR-196a, and this has been linked to poor patient outcome, with similar effects seen in NSCLC and gastric cancer [16,25]. Liu et al showed overexpression of miR-196a in HNSCC cells had little effect on tumour cell proliferation, but did result in an increase in cell migration [22], which is in keeping with our findings and also those seen on manipulation of miR-196a expression in a number of other cancer cell types [46]. Conversely, overexpression of miR-196a in NOKs resulted in a marked reduction in proliferation [30], but it is difficult to directly compare these effects with those seen in HNSCC cells given the markedly different genomic and transcriptomic background. Our investigations of transfection of pre-miR-196a into the immortalised NOK cell line OKF4 showed no change in proliferation and a small increase in migration (data not shown).

*HOXB9* expression is elevated in a range of cancers including breast, NSCLC and hepatocellular carcinoma [5,7,47], but decreased expression has been related to poor prognosis in others [48]. The increased expression of HOXB9 in HNSCC which we have demonstrated *in vitro* and in tissues has also been shown in a small number of other microarray analyses and in comparable qPCR assessment of *HOX* gene expression in HNSCC [17,49,50]. The identification of the differential expression of *HOXB9* in HNSCC in the array analysis of Ginos et al [49] is notable as *HOXB9* was not identified in the original Hunter et al dataset [3], but was shown in this study by qPCR (Fig 2). This illustrates the limitations of many expression array analyses given the variability of samples analysed and uncertainties surrounding the probesets which have been used to detect the targets. For the first time we have demonstrated increased *HOXB9* expression in OPM cultures, which was not seen in the investigation of Hassan et al [17]. However, 3 of the 4 cultures we used were derived from severely dysplastic lesions which progressed to HNSCC in less than 6 months (D19, D20 and D35). This indicates that a more detailed analysis of the expression and role of HOXB9 in OPM is warranted to determine if expression of HOXB9 increases with OPM severity.

We have shown that *HOXB9* enhances migration, invasion and proliferation of both HNSCC and OPM cells in keeping with the effects of high *HOXB9* expression in other cancers, but which has not been demonstrated previously in HNSCC or in pre-invasive disease. Our findings are in keeping with the observed increase in migration and invasion in breast cancer, where elevated *HOXB9* expression enhances the DNA damage response, not only conferring resistance to ionizing radiation, but also associated with induction of epithelial to mesenchymal transition (EMT) [51]. Investigations in other cancers have demonstrated similar roles in addition to enhancement of tumour proliferation and angiogenesis [13,52]. These are important issues to address in relation to both miR196a and HOXB9 and we are currently undertaking further investigation of the mechanisms of altered migration and invasion.

The apparent similarity in the patterns of expression of *HOXB9* and miR-196a has been demonstrated in Hodgkin lymphoma and AML cells, suggesting co-regulation, but in neither case were further investigations conducted [53,54]. The presence of numerous polycistronic transcripts from the *HOX* loci has been inferred from analysis of high resolution transcriptional profiling, including a putative transcript which includes *HOXB7*, *HOXB8*, *HOXB9* and miR-196a-1 [55]. This transcript is annotated in Ensembl (Transcript: RP11-357H14.19–001), without direct experimental evidence of its existence. We have demonstrated the existence of a transcript that at least spans the 6.3kb region between the open reading frames of *HOXB9* and miR-196a-1. However, what is not clear is what proportion of *HOXB9* and miR-196a expression is accounted for by this transcript. Given that the expression of *HOXB9* and miR-196a is not completely coordinated, particularly in OPM cells (Figs 2B and 1C), it is likely that this transcript only contributes a proportion of the pool of processed mRNA for *HOXB9* and miR-196a, indicating that, whilst there may be co-expression, expression of HOXB9 and miR-196a is under the control of other factors, which remain to be elucidated, which are responsible for different transcripts. An understanding of the primary transcripts used in the expression of both *HOXB9* and miR-196a will be important in directing possible approaches to inhibition.

There are significant difficulties in the identification of endogenous miRNA targets and this has led to the use of a wide range of techniques, ranging from *in-silico* methods to direct assessment of binding between the miRNA seed sequence and binding site in the 3’UTR of cognate mRNAs. We decided to pursue novel targets of miR-196a by expression microarray following miR-196a over-expression and knockdown, despite the acknowledged limitations of this approach, including possible effects on protein and not mRNA. The microarray expression data and subsequent *in-silico* analysis identified a number of potential targets of miR-196a, including some which have been identified in other cell types, such as *HOXC8* (S3 Fig) [18]. However, the overall GO enrichment analysis only identified a small number of enriched biological processes that were affected by manipulation of miR-196a expression. The most enriched categories included DNA repair, the DNA damage response and various transporter pathways (S1 Table). These do not appear to map directly to the observed phenotype, which may be partly due to the relatively small number of differentially expressed genes identified and also demonstrate the interaction of miR-196a in a controlling a wide range of different processes in the cell. Interestingly, we observed no consistent changes in the expression of miR-196a targets which have been identified in other cancer types. This may indicate a level of tumour specificity in the actions of miR-196a.

Overall, the highest ranked novel gene target in our analysis was MAMDC2. The level of MAMDC2 expression varies between the NOK, OPM and HNSCC cells, indicative of control of expression by other, as yet unknown mechanisms. However, given the effect on transfection of pre-miR196 or anti-miR196 presented in Fig 6C and 6D, there is good evidence that MAMDC2 is under the direct control of miR196a. MAMDC2 is a transmembrane cell adhesion protein of the immunoglobulin superfamily. MAM (meprin/A5-protein/PTPmu) domains are present in numerous proteins with varied functions, with many associated with promotion of cell-cell adhesion [56,57]. Correspondingly, mutations in the MAM domain of the receptor protein tyrosine phosphatase T have been shown to promote cancer cell migration and metastasis in colorectal cancer [58,59]. Whilst this would be in keeping with the phenotype observed upon transfection with anti-miR-196a, this requires further investigation to establish if this observation can be attributed to the biological functions of MAMDC2. Given that a number of small molecules which interfere with cell adhesion are currently being tested as anticancer agents, this may also represent a novel therapeutic target.

Given that elevated *HOXB9* expression and high serum levels of miR-196a have been associated with poor prognosis and recurrence in other tumour types [5,7,16], it is conceivable, that together, they may be of prognostic use in HNSCC. Potential therapeutic applications may be more challenging as, whilst inhibitors of the *HOX-PBX* interaction are available and effective [8], some of the 5’ *HOX* genes (e.g. *HOXD10* and *HOXB9*) are less PBX dependent which may limit their use [60], thus new strategies for disruption of this interaction may prove more effective. Furthermore, if the molecular mechanisms responsible for the transcription of the novel primary transcript identified here can be elucidated, it may be possible to inhibit its expression and subsequent effects.

## Conclusions

In this manuscript we have demonstrated that both HOXB9 and miR196a are highly expressed in HNSCC both *in vivo* and *in vitro*. Both exert a pro-tumour phenotype and we have demonstrated a putative primary transcript which bears both coding sequences. This suggests that inhibition of expression of genes across this locus may prove a useful therapeutic strategy as this will inhibit a number of pro-tumorigenic factors. Furthermore we have identified MAMDC2 as a novel target of miR196a in HNSCC, suggesting further interrogation of the biological significance of the miR-196a/MAMDC2 axis may enhance understanding of HNSCC pathogenesis.

## Supporting Information

S1 Fig Schematic diagram of the putative primary transcript and the length of the PCR product for both the primers utilised in the nested PCR analysis. (TIF)

S2 Fig Expression of all HOX genes analysed by qPCR, showing data in the full panel of cell cultures tested, including normal (black), OPM (red) and HNSCC (blue), arranged by group (A-D) and in numerical order. (TIF)

S3 Fig Expression of putative miR196a targets suggested form investigation in other cancers, as assessed by qPCR in anti-miR196a transfected D19 and B16 cells, and pre-miR 196a transfected OKF4 cells in panel D. **A**: Keratin V; **B**: Anexin A1; **C** S100A9; **D**: HOXC8. Only HOXC8 shows significant changes in expression and this only in D19 (p<0.01). The data does not support regulation of KRT5, ANXA1 or S100A9 by miR196a in HNSCC.
(TIF)

S1 Table Clinical details of the HNSCC and OPM cell lines used in this study. All cell lines are HPV negative.
(DOCX)

S2 Table Clinical and pathological details of the 25 HNSCC samples in the TMA used for HOXB9 IHC. FOM = Floor of mouth, RM = retromolar, BM = buccal mucosa.
(DOCX)

S3 Table Clinical pathological details of HNSCC samples used for Laser capture microdissection FOM = Floor of mouth, RM = retromolar, BM = buccal mucosa. (DOCX)

S4 Table A full list of qPCR primers used in this study. (DOCX)

S5 Table Gene ontology enrichment analysis demonstrating significantly enriched GO biological processes on manipulation of miR-196a expression. The total number of significantly differentially expressed genes entered into this analysis was 353. Analysis generated by analysis of the gene list in DAVID (http://david.abcc.ncifcrf.gov).
(DOCX)
