# Supplementary figures and images for: The Role of HOXB9 and miR-196a in Head and Neck Squamous Cell Carcinoma
Source: PLoS One. 2015 Apr 10;10(4):e0122285. doi: 10.1371/journal.pone.0122285 (PMC4393232; doi:10.1371/journal.pone.0122285)

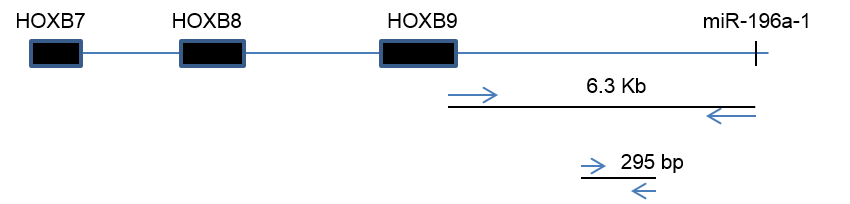

Supplement: S1 Fig — (TIF) [file pone.0122285.s001.tif]

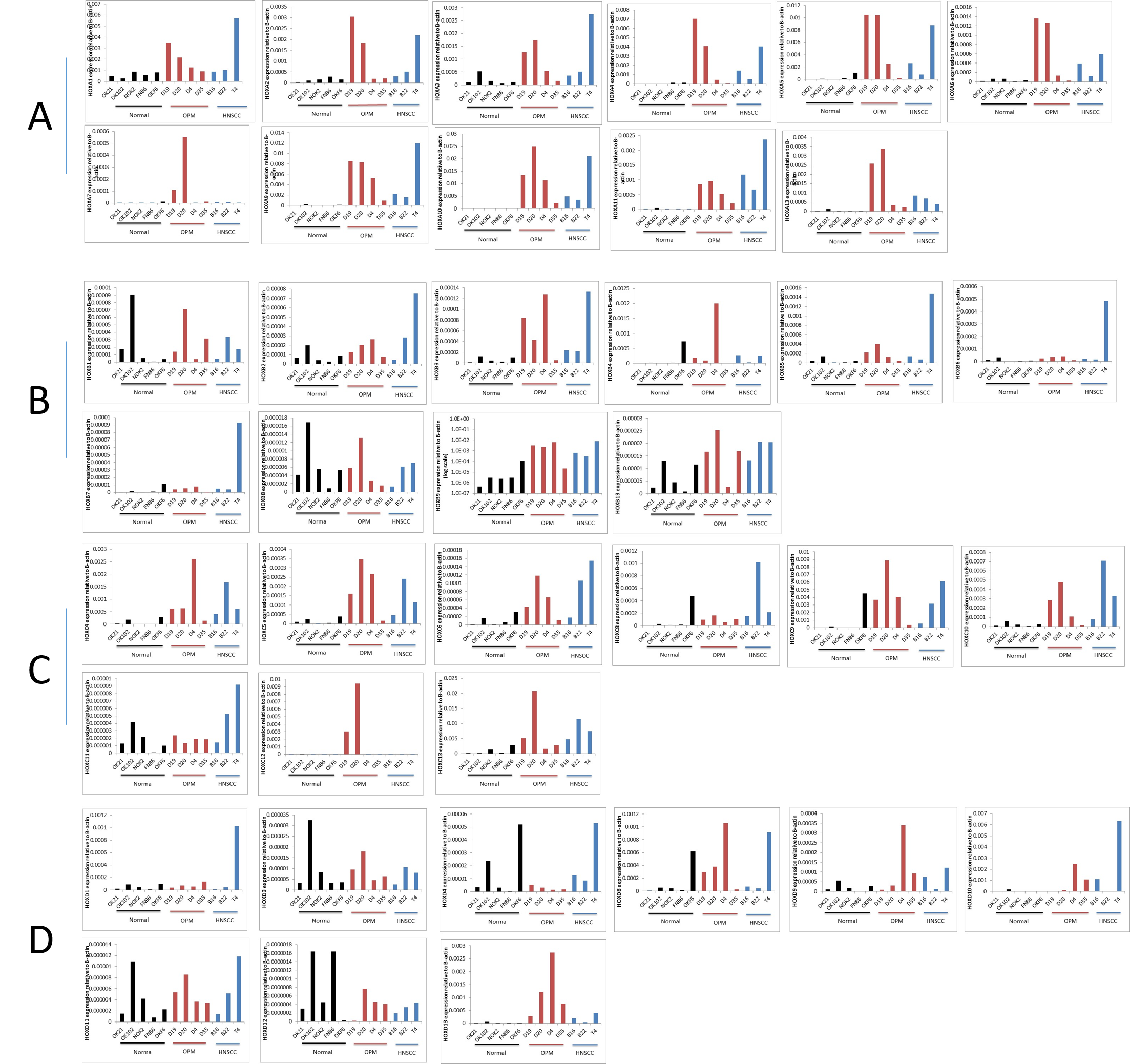

Supplement: S2 Fig — (TIF) [file pone.0122285.s002.tif]

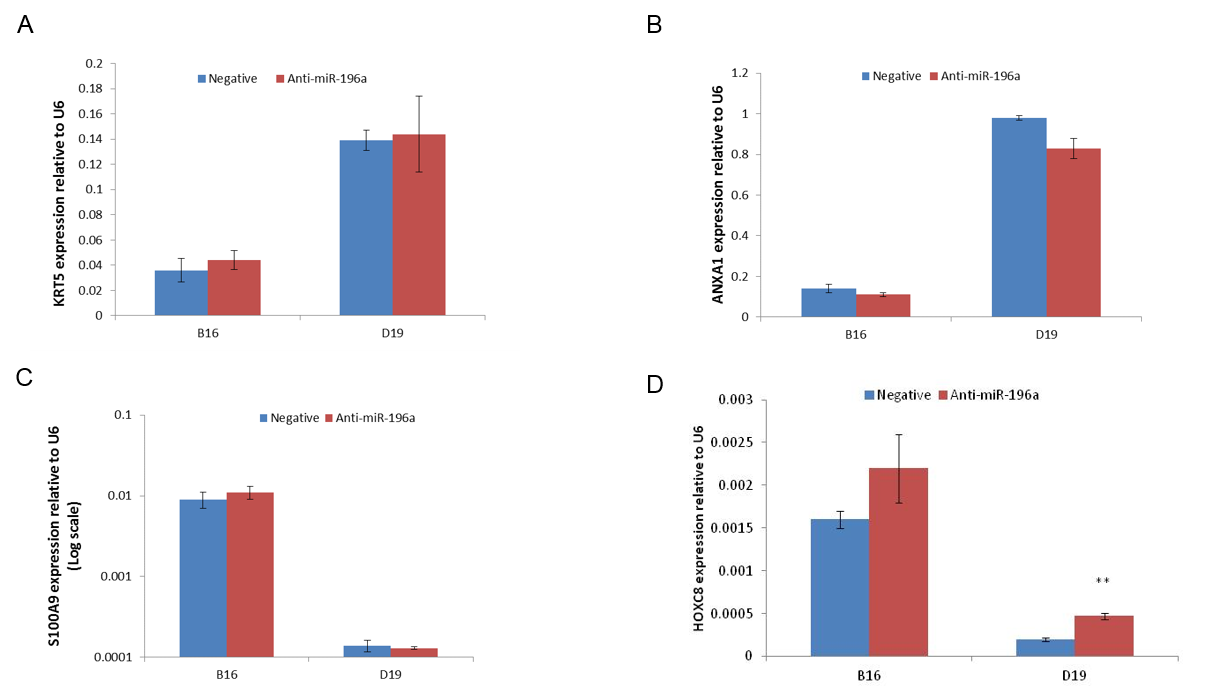

Supplement: S3 Fig — A: Keratin V; B: Anexin A1; C S100A9; D: HOXC8. Only HOXC8 shows significant changes in expression and this only in D19 (p<0.01). The data does not support regulation of KRT5, ANXA1 or S100A9 by miR196a in HNSCC. (TIF) [file pone.0122285.s003.tif]
