# Supplementary material for: The Role of HOXB9 and miR-196a in Head and Neck Squamous Cell Carcinoma
Source: PLoS One. 2015 Apr 10;10(4):e0122285. doi: 10.1371/journal.pone.0122285 (PMC4393232; doi:10.1371/journal.pone.0122285)
Supplement: S1 Table — All cell lines are HPV negative. (DOCX) [file pone.0122285.s004.docx]

| **Cell line** | **Primary lesion site** | **Stage** | **Source of cell line** | **Reference** |
| --- | --- | --- | --- | --- |
| B16 | Lateral Tongue | T2 N0 | Recurrent tumour | 31, 32 |
| B22 | Lateral Tongue | T4 N3 | LN metastasis | 31, 32 |
| B56 | Lateral Tongue | T4 N1 | Primary tumour | 31, 32 |
| T4 | Floor of mouth | T4 N0 | Primary tumour | 34, 35 |
| H357 | Lateral Tongue | T1 N0 | Primary tumour | 33 |
| D19 | Lateral tongue | Severe dysplasia | N/A | 34, 35 |
| D20 | Lateral tongue | Moderate dysplasia | N/A | 34, 35 |
| D4 | Ventral tongue | Moderate dysplasia | N/A | 34, 35 |
| D35 | Ventral tongue | Severe dysplasia | N/A | 34, 35 |

**Table S1.**
