# Supplementary material for: The Role of HOXB9 and miR-196a in Head and Neck Squamous Cell Carcinoma
Source: PLoS One. 2015 Apr 10;10(4):e0122285. doi: 10.1371/journal.pone.0122285 (PMC4393232; doi:10.1371/journal.pone.0122285)
Supplement: S2 Table — FOM = Floor of mouth, RM = retromolar, BM = buccal mucosa. (DOCX) [file pone.0122285.s005.docx]

| **Case** | **Age/Gender** | **Diagnosis** | **Site** |
| --- | --- | --- | --- |
| 1 | 68M | SCC, Moderately differentiated | Tongue |
| 2 | 67F | SCC, Moderately differentiated | FOM |
| 3 | 48M | SCC, Moderately differentiated | FOM |
| 4 | 51M | SCC, Moderately differentiated | Tongue |
| 5 | 52M | SCC, Moderately differentiated | RM |
| 6 | 51M | SCC, Moderately differentiated | RM |
| 7 | 44M | SCC, Moderately differentiated | Tongue |
| 8 | 64F | SCC, Moderately differentiated | Tongue |
| 9 | 47M | SCC, Well differentiated | Tongue |
| 10 | 76F | SCC, Moderately differentiated | Tongue |
| 11 | 40F | SCC, Moderately differentiated | Tongue |
| 12 | 73M | SCC, Well differentiated | Tongue |
| 13 | 80M | SCC, Well differentiated | Gingiva |
| 14 | 52F | SCC, Moderately differentiated | RM |
| 15 | 79M | SCC, Well differentiated | Palate |
| 16 | 46F | SCC, Moderately differentiated | Tongue |
| 17 | 77M | SCC, Poorly differentiated | Tongue |
| 18 | 65M | SCC, Moderately differentiated | Palate |
| 19 | 62M | SCC, Moderately differentiated | FOM |
| 20 | 63M | SCC, Well differentiated | RM |
| 21 | 74F | SCC, Moderately differentiated | FOM |
| 22 | 69M | SCC, Moderately differentiated | Gingiva |
| 23 | 64M | SCC, Moderately differentiated | Gingiva |
| 24 | 47M | SCC, Moderately differentiated | BM |
| 25 | 36M | SCC, Well differentiated | Tongue |

Table S2.
