# Supplementary material for: The Role of HOXB9 and miR-196a in Head and Neck Squamous Cell Carcinoma
Source: PLoS One. 2015 Apr 10;10(4):e0122285. doi: 10.1371/journal.pone.0122285 (PMC4393232; doi:10.1371/journal.pone.0122285)
Supplement: S3 Table — (DOCX) [file pone.0122285.s006.docx]

| **Case** | **Age/Gender** | **Diagnosis** | **Site** |
| --- | --- | --- | --- |
| 1 | 52F | SCC, Moderately differentiated | Tongue |
| 2 | 59M | SCC, Moderately differentiated | Tongue |
| 3 | 71M | SCC, Moderately differentiated | RM |
| 4 | 68F | SCC, Moderately differentiated | Gingiva |
| 5 | 65F | SCC, Moderately differentiated | Palate |
| 6 | 90M | SCC, Moderately differentiated | BM |
| 7 | 79F | SCC, Moderately differentiated | BM |
| 8 | 83F | SCC, Poorly differentiated | Gingiva |
| 9 | 58F | SCC, Poorly differentiated | FOM |
| 10 | 72F | SCC, Poorly differentiated | Tongue |
| 11 | 53M | SCC, Moderately differentiated | BM |
| 12 | 53M | SCC, Poorly differentiated | Gingiva |
| 13 | 85F | SCC, Moderately differentiated | Tongue |
| 14 | 56M | SCC, Moderately differentiated | Tongue |
| 15 | 71M | SCC, Moderately differentiated | Gingiva |
| 16 | 72F | SCC, Poorly differentiated | BM |

Table S3.
