# Supplementary material for: The Role of HOXB9 and miR-196a in Head and Neck Squamous Cell Carcinoma
Source: PLoS One. 2015 Apr 10;10(4):e0122285. doi: 10.1371/journal.pone.0122285 (PMC4393232; doi:10.1371/journal.pone.0122285)
Supplement: S4 Table — (DOCX) [file pone.0122285.s007.docx]

| **HOX gene name** | **Product length (bp)** | **Primer sequence** |
| --- | --- | --- |
| HOXA1 | 153 | F: 5’ CTGGCCCTGGCTACGTATAA 3’ R: 5’ TCCAACTTTCCCTGTTTTGG 3’ |
| HOXB1 | 157 | F: 5’ TTCAGCAGAACTCCGGCTAT 3’ R: 5’ CCTCCGTCTCCTTCTGATTG 3’ |
| HOXD1 | 232 | F: 5’ TTCAGCACCAAGCAACTGAC 3’  R: 5’ TAGTGGGGGTTGTTCCAGAG 3’ |
| HOXA2 | 176 | F: 5’ TTCAGCAAAATGCCCTCTCT 3’  R: 5’ TAGGCCAGCTCCACAGTTCT 3’ |
| HOXB2 | 258 | F: 5’ CTCCCAAAATCGCTCCATTA 3’  R: 5’ GAAAGGAGGAGGAGGAGGAA 3’ |
| HOXA3 | 227 | F: 5’ ACCTGTGATAGTGGGCTTGG 3’  R: 5’ ATACAGCCATTCCAGCAACC 3’ |
| HOXB3 | 299 | F: 5’ TATGGCCTCAACCACCTTTC 3’ R: 5’ AAGCCTGGGTACCACCTTCT 3’ |
| HOXD3 | 176 | F: 5’ CAGCCTCCTGGTCTGAACTC 3’  R: 5’ ATCCAGGGGAAGATCTGCTT 3’ |
| HOXA4 | 271 | F: 5’ CCCTGGATGAAGAAGATCCA 3’ R: 5’ AATTGGAGGATCGCATCTTG 3’ |
| HOXB4 | 155 | F: 5’ TCTTGGAGCTGGAGAAGGAA 3’  R: 5’ GTTGGGCAACTTGTGGTCTT 3’ |
| HOXC4 | 276 | F: 5’ CGCTCGAGGACAGCCTATAC 3’  R: 5’ GCTCTGGGAGTGGTCTTCAG 3’ |
| HOXD4 | 173 | F: 5’ TCAAATGTGCCATAGCAAGC 3’  R: 5’ TCCATAGGGCCCTCCTACTT 3’ |
| HOXA5 | 193 | F: 5’ CCGGAGAATGAAGTGGAAAA 3’  R: 5’ ACGAGAACAGGGCTTCTTCA 3’ |
| HOXB5 | 189 | F: 5’ AAGGCCTGGTCTGGGAGTAT 3’  R: 5’ GCATCCACTCGCTCACTACA 3’ |
| HOXC5 | 268 | F: 5’ CAGTTACACGCGCTACCAGA 3’  R: 5’ AGAGAGGAAAGGCGAAAAGG 3’ |
| HOXA6 | 158 | F: 5’ AAAGCACTCCATGACGAAGG 3’  R: 5’ TCCTTCTCCAGCTCCAGTGT 3’ |
| HOXB6 | 184 | F: 5’ ATTTCCTTCTGGCCCTCACT 3’  R: 5’ GGAAGGTGGAGTTCACGAAA 3’ |
| HOXC6 | 190 | F: 5’ AAGAGGAAAAGCGGGAAGAG 3’  R: 5’ GGTCCACGTTTGACTCCCTA 3’ |
| HOXA7 | 285 | F: 5’ TGGTGTAAATCTGGGGGTGT 3’  R: 5’ TCTGATAAAGGGGGCTGTTG 3’ |
| HOXB7 | 249 | F: 5’ CAGCCTCAAGTTCGGTTTTC 3’  R: 5’ CGGAGAGGTTCTGCTCAAAG 3’ |
| HOXB8 | 265 | F: 5’ GTAGGCTTCAGCTGGGACTG 3’  R: 5’ GGGAGCCTTTGCTTAAATCC 3’ |
| HOXC8 | 150 | F: 5’ CTCAGGCTACCAGCAGAACC 3’  R: 5’ TTGGCGGAGGATTTACAGTC 3’ |
| HOXD8 | 290 | F: 5’ TCAAATGTTTCCGTGGATGA 3’  R: 5’ GCTCTTGGGCTTCCTTTTTC 3’ |
| HOXA9 | 203 | F: 5’ AATAACCCAGCAGCCAACTG 3’ R: 5’ ATTTTCATCCTGCGGTTCTG 3’ |
| HOXB9 | 198 | F: 5’ TAATCAAAGACCCGGCTACG 3’  R: 5’ CTACGGTCCCTGGTGAGGTA 3’ |
| HOXC9 | 190 | F: 5’ AGACGCTGGAACTGGAGAAG 3’  R: 5’ AGGCTGGGTAGGGTTTAGGA 3’ |
| HOXD9 | 236 | F: 5’ TCCCCCATGTTTCTGAAAAG 3’  R: 5’ GGGCTCCTCTAAGCCTCACT 3’ |
| HOXA10 | 159 | F: 5’ ACACTGGAGCTGGAGAAGGA 3’  R: 5’ GATCCGGTTTTCTCGATTCA 3’ |
| HOXC10 | 289 | F: 5’ CGCCTGGAGATTAGCAAGAC 3’  R: 5’ GGTCCCTTGGAAGGAGAGTC 3’ |
| HOXD10 | 154 | F: 5’ GCTCCTTCACCACCAACATT 3’  R: 5’ AAATATCCAGGGACGGGAAC 3’ |
| HOXA11 | 279 | F: 5’ CGCTGCCCCTATACCAAGTA 3’  R: 5’ GTCAAGGGCAAAATCTGCAT 3’ |
| HOXC11 | 186 | F: 5’ CGGAACAGCTACTCCTCCTG 3’  R: 5’ CAGGACGCTGTTCTTGTTGA 3’ |
| HOXD11 | 253 | F: 5’ GGGGCTACGCTCCCTACTAC 3’  R: 5’ GCTGCCTCGTAGAACTGGTC 3’ |
| HOXC12 | 180 | F: 5’ CAAGCCCTATTCGAAGTTGC 3’  R: 5’ GCTTGCTCCCTCAACAGAAG 3’ |
| HOXD12 | 201 | F: 5’ CGCTTCCCCCTATCTCCTAC 3’  R: 5’ CTTCGGGCGCATAGAACTTA 3’ |
| HOXA13 | 176 | F: 5’ GGATATCAGCCACGACGAAT 3’  R: 5’ ATTATCTGGGCAAAGCAACG 3’ |
| HOXB13 | 234 | F: 5’ CTTGGATGGAGCCAAGGATA 3’  R: 5’ CCGCCTCCAAAGTAACCATA 3’ |
| HOXC13 | 170 | F: 5’ GTGGAAATCCAAGGAGGACA 3’  R: 5’ TTGTTGAGGGACCCACTCTC 3’ |
| HOXD13 | 265 | F: 5’ GGGGATGTGGCTCTAAATCA 3’  R: 5’ AACCTGGACCACATCAGGAG 3’ |
| β-actin | 182 | F: 5’ ATGTACCCTGGCATTGCCGAC 3’  R: 5’ GACTCGTCATACTCCTGCTTG 3’ |
| MAMDC2 | 187 | F: 5’ ATGCTGTTAAGGGGCGTCCTCCTG 3’  R: 5’ CCTGCTTGCCAAAGGAGGTATCCA 3’ |

**Table S4**.
