# Supplementary material for: The Role of HOXB9 and miR-196a in Head and Neck Squamous Cell Carcinoma
Source: PLoS One. 2015 Apr 10;10(4):e0122285. doi: 10.1371/journal.pone.0122285 (PMC4393232; doi:10.1371/journal.pone.0122285)
Supplement: S5 Table — The total number of significantly differentially expressed genes entered into this analysis was 353. Analysis generated by analysis of the gene list in DAVID (http://david.abcc.ncifcrf.gov). (DOCX) [file pone.0122285.s008.docx]

| Term | Count | % | P Value | Genes | Fold Enrich-ment |
| --- | --- | --- | --- | --- | --- |
| GO:0033554: Cellular response to stress | 16 | 0.78 | 0.002 | *C9ORF102, ZAK, SMC5, SIRT7, FOXO3, HSPA1L, RFC3, DCLRE1B, PSEN1, STAC, BTG2, GSK3B, MUS81, EIF2B3, FANCA, ERCC1* | 2.5 |
| GO:0015837: Amine transport | 7 | 0.34 | 0.002 | *SLC1A4, SLC6A9, PSEN1, SLC38A7, SLC36A4, CLN8, CRYM* | 5.2 |
| GO:0006974: Response to DNA damage stimulus | 12 | 0.59 | 0.004 | *HSPA1L, RFC3, C9ORF102, DCLRE1B, PSEN1, ZAK, BTG2, MUS81, SMC5, FOXO3, FANCA, ERCC1* | 2.8 |
| GO:0046942: Carboxylic acid transport | 7 | 0.34 | 0.007 | *SLC1A4, SLC6A9, PSEN1, SLC38A7, ABCC3, SLC36A4, CLN8* | 4.2 |
| GO:0006281: DNA repair | 9 | 0.44 | 0.016 | *HSPA1L, RFC3, C9ORF102, DCLRE1B, BTG2, MUS81, SMC5, FANCA, ERCC1* | 2.8 |
| GO:0045892: Negative regulation of transcription, DNA-dependent | 10 | 0.49 | 0.020 | *HOXC8, SBNO2, EHMT1, ARID4A, E2F6, SIRT7, SKIL, ZNF254, DNAJB6, CRYM* | 2.5 |
| GO:0010558: Negative regulation of macromolecule biosynthetic process | 13 | 0.64 | 0.022 | *SBNO2, EHMT1, ARID4A, E2F6, CENPF, SIRT7, ZNF254, EIF4EBP1, HOXC8, SKIL, EIF2B3, CRYM, DNAJB6* | 2.1 |
| GO:0021532: Neural tube patterning | 3 | 0.15 | 0.023 | *PSEN1, GBX2, RPGRIP1L* | 12.5 |
| GO:0009612: Response to mechanical stimulus | 4 | 0.20 | 0.026 | *BTG2, MGP, DNAH1, TIMP3* | 6.2 |
| GO:0010629: Negative regulation of gene expression | 12 | 0.59 | 0.029 | *HOXC8, SBNO2, EHMT1, ARID4A, E2F6, BNIP3L, CENPF, SIRT7, SKIL, ZNF254, DNAJB6, CRYM* | 2.1 |
| GO:0032318: Regulation of Ras GTPase activity | 5 | 0.24 | 0.031 | *FICD, SH3D20, C6ORF170, EVI5L, TBC1D20* | 4.2 |

**Table S5.**
